# Alzheimer’s disease and microbiota: The MICMALZ cohort

**DOI:** 10.1002/alz.088855

**Published:** 2025-01-09

**Authors:** Karim Bennys

**Affiliations:** ^1^ Memory Resources and Research Center, CHRU Gui de Chauliac, Montpellier France

## Abstract

**Background:**

Microbiota is modulated by normal aging, but also by Alzheimer’s disease (AD) risk factors as poor diet or alteration of sleep patterns. Patients with AD exhibit a dysbiosis characterized by changes in the relative proportions of specific bacterial phyla. Eventually, fecal microbiota transplants (FMT) can improve cognitive deficits and reduce amyloid‐ß deposition, at least in mouse models of AD.

**Method:**

We generated a cohort of AD patients, with control participants matching on age, sex, body mass index, Mini Nutritional Assessment® and education, to sample and compare microbiota composition in the stool and blood compartments. This metagenomic study will be completed by targeted and non‐targeted metabolomic analysis to inform about microbiota impact on the host. We precisely evaluated cognition, sleep parameters and dietary habits of the subjects. Moreover, stool samples from 19 patients were pooled by 4, with similar age and sex, from each group, and were transplanted in a mouse model of AD (5XFAD, n = 94) or their control littermates (n = 113) to evaluate the consequences on gut microbiota composition, memory‐related behaviors and molecular and cellular biomarkers of AD physiopathology.

**Result:**

We generated a cohort of well characterized patients representative of mild to moderate AD patients with 45 AD patients [age 75 (SD 0.9), 51% women, minimental state examination (MMSE) 22 (interquartile range, IQR, 17‐25)] and 37 controls [age 72 (SD 1.5), 62% women, MMSE 29 (IQR 27‐30)]. Our preliminary results indicate i) a potential dysbiosis in AD patients that translates to mouse microbiota composition following FMT. A specific bacterial genus is increased both in 5XFAD and control mice potentially indicating over‐representation in AD patients relative to the controls; ii) an impact of microbiota transplanted from AD patients to mouse model on memory and behavior, with an alteration of novel object recognition but also on biomarkers of AD pathology including an increase in mouse Aß1‐42 expression level.

**Conclusion:**

Our results suggest that gut microbiota dysbiosis is associated with AD status and point to a specific bacterial genus. Moreover, FMT from AD patients in an AD mouse model recapitulates important features of the disease, with memory impairment and Aß1‐42 accumulations.

